# Intelligent Counter-UAV Threat Detection Using Hierarchical Fuzzy Decision-Making and Sensor Fusion

**DOI:** 10.3390/s25196091

**Published:** 2025-10-02

**Authors:** Fani Arapoglou, Paraskevi Zacharia, Michail Papoutsidakis

**Affiliations:** Department of Industrial Design and Production Engineering, University of West Attica, Egaleo, 12241 Athens, Greece; mscdrones8096603@uniwa.gr (F.A.); mipapou@uniwa.gr (M.P.)

**Keywords:** Counter-UAV systems, sensor fusion, hierarchical fuzzy logic, decision-making, UAV threat detection, surveillance systems

## Abstract

**Highlights:**

This study presents a hierarchical fuzzy decision-making approach for detecting UAV threats using data from multiple sensors. It introduces a three-level fuzzy system that evaluates sensor performance and selects the best sensor combinations to improve threat detection in Counter-UAV operations.

**What are the main findings?**

- A hierarchical fuzzy inference framework was developed to evaluate and optimize multi-sensor configurations for Counter-UAV applications.
- The system demonstrates that combining fuzzy logic with sensor fusion improves detection accuracy while balancing cost, range, and operational effectiveness.

**What is the implication of the main finding?**

- The proposed approach enables intelligent and adaptive sensor selection in real-time threat environments, enhancing decision-making in Counter-UAV systems.
- The modular and interpretable framework can be applied to other domains requiring uncertainty management and multisensor optimization.

**Abstract:**

This paper proposes an intelligent hierarchical fuzzy decision-making framework for threat detection and identification in Counter-Unmanned Aerial Vehicle (Counter-UAV) systems, based on the fusion of heterogeneous sensor data. To address the increasing complexity and ambiguity in modern UAV threats, this study introduces a novel three-stage fuzzy inference architecture that supports adaptive sensor evaluation and optimal pairing. The proposed methodology consists of three-layered Fuzzy Inference Systems (FIS): FIS-A quantifies sensor effectiveness based on UAV flight altitude and detection probability; FIS-B assesses operational suitability using sensor range and cost; and FIS-C synthesizes both outputs, along with sensor capability overlap, to determine the composite suitability of sensor pairs. This hierarchical structure enables detailed analysis and system-level optimization, reflecting real-world constraints and performance trade-offs. Simulation-based evaluation using diverse sensor modalities (EO/IR, Radar, Acoustic, RF), supported by empirical data and literature, demonstrates the framework’s ability to handle uncertainty, enhance detection reliability, and support cost-effective sensor deployment in Counter-UAV operations. The framework’s modularity, scalability, and interpretability represent significant advancements in intelligent Counter-UAV system design, offering a transferable methodology for dynamic threat environments.

## 1. Introduction

Unmanned Aerial Vehicles (UAVs) have rapidly proliferated in recent years, offering diverse applications across commercial, industrial, and recreational domains. However, their increasing accessibility has also led to their exploitation in hostile contexts, such as unauthorized surveillance, contraband delivery, and disruption of critical infrastructure [1,2]. This dual-use nature of UAVs poses significant security challenges for law enforcement, defense, and infrastructure protection agencies worldwide.

Counter-UAV systems have emerged as a critical component of modern airspace security. These systems rely on various sensing modalities—such as radar, radio frequency (RF) analysis, electro-optical/infrared (EO/IR) imaging, and acoustic detection—to monitor, track, and classify aerial threats [3,4,5,6,7,8,9]. While each sensor technology offers specific advantages, they are also subject to inherent limitations. For example, radar systems may struggle with small cross section targets in cluttered environments [10], EO/IR systems are heavily influenced by lighting and weather [6,7], acoustic sensors are range-limited [8,9], and RF detection is ineffective against autonomous UAVs with no active communication links [10,11].

Recent studies have explored various approaches to UAV threat detection using multi-sensor systems. Shi et al. [12] demonstrated a practical anti-drone platform combining RF, EO, and acoustic sensors, highlighting the benefits and limitations of each modality. Park et al. [3] and Wang et al. [4] provided comprehensive surveys on Counter-UAV architectures and emphasized the need for intelligent, adaptive decision-making frameworks. Yan et al. [5] focused on passive sensor challenges in urban environments, reinforcing the importance of robust fusion strategies. Besada et al. [13] further compared sensor types for unmanned traffic management, underscoring the trade-offs in range, cost, and reliability.

Traditionally, many Counter-UAV deployments have relied on single-sensor systems or simple rule-based data fusion approaches [4,12,14]. However, these methods often fail to capture the complex, uncertain, and dynamic nature of real-world UAV encounters, especially in urban environments where signal interference, occlusions, and multi-path effects can degrade performance [5,6,7]. Moreover, the process of selecting optimal sensor combinations for different operational scenarios remains a challenging task, particularly when multiple performance criteria must be considered simultaneously.

Conventional fusion systems are typically rigid, requiring manual adjustment of rules for each new scenario or sensor type, which limits their scalability and adaptability. Similarly, single-layer fuzzy systems, while more flexible, often have limited capacity to represent the multi-dimensional trade-offs and interactions among diverse sensor modalities. As the number of sensors and operational criteria increases, these approaches can become unwieldy, less interpretable, and difficult to maintain or extend. This restricts their effectiveness in dynamic and complex threat environments, where rapid adaptation and transparent decision-making are essential.

Recent advances in computational intelligence, including fuzzy logic (FL) and multi-criteria decision-making (MCDM), have shown promise in addressing these challenges. Fuzzy logic, in particular, offers a robust mathematical framework for handling uncertainty and imprecision in sensor measurements and operational assessments [15,16,17]. Despite this potential, there is limited research on applying hierarchical fuzzy inference systems (FIS) to systematically evaluate and rank Counter-UAV sensor configurations across varying mission requirements.

FL has been increasingly applied in UAV-related domains to manage uncertainty and enhance decision-making. Recent research has explored the use of fuzzy logic in UAV detection, control, and sensor fusion systems. Wu et al. [18] developed a fuzzy decision model to evaluate multi-sensor confidence levels for UAV target detection, highlighting the benefits of structured uncertainty modeling. Cook and Cohen [19] proposed a fuzzy-based sensor confidence system for real-time fusion of GPS, radar, and onboard sensors in small UAS operations, emphasizing adaptability under uncertain conditions. Singh et al. [20] provided a comprehensive review of fuzzy mathematical algorithms in autonomous vehicles and drones, emphasizing their role in decision-making and sensor fusion. Dumitrescu et al. [21] introduced a multi-sensor UAV detection framework integrating acoustic, EO/IR, radar, and RF data with AI-based classification, addressing the challenges of threat identification in restricted environments. Hao et al. [22] proposed a fuzzy preference-based fusion method for multi-sensor systems, demonstrating improved adaptability and accuracy in noisy conditions. Beyond threat detection, Tsitses et al. [23] introduced a fuzzy-based system for autonomous UAV ship deck landing, showcasing the versatility of fuzzy inference in dynamic and constrained environments. These studies collectively underscore the growing relevance of fuzzy logic in UAV-related decision-making and motivate the hierarchical approach proposed in this work, which focuses on intelligent sensor selection and fusion for Counter-UAV operations.

Building on these insights, this study presents a hierarchical fuzzy inference system designed to evaluate sensor combinations under realistic operational constraints. The proposed framework addresses the identified gap by introducing a structured decision-making approach specifically designed for Counter-UAV applications. It consists of three subsystems: FIS-A assesses sensor effectiveness under operational conditions, such as flight altitude and detection probability; FIS-B evaluates operational suitability using sensor range and cost; FIS-C integrates both, along with empirically derived sensor-pair overlap values. Two scenarios were used for validation—one simulating balanced, low-cost deployments with high detection reliability, and another reflecting more demanding, long-range operations with higher costs and environmental constraints. The results show that the proposed framework effectively captures trade-offs between sensor technologies, supports informed decision-making, and emphasizes the value of sensor complementarity in multi-sensor Counter-UAV configurations.

The proposed approach involves the development of an intelligent control system utilizing fuzzy logic. The key contributions of this work can be outlined in the following main aspects:

- It introduces a hierarchical fuzzy inference system specifically designed for sensor selection and pairing in Counter-UAV applications. This multi-layered approach enables interpretable, scenario-adaptive, and operationally realistic decision-making, addressing the limitations of traditional systems, which often lack scalability and adaptability.
- It uses an empirical sensor pair metric, which quantifies the complementarity and redundancy between sensor types. To our knowledge, this metric is integrated into the fuzzy decision process for the first time in the context of Counter-UAV sensor fusion, enabling more effective and transparent optimization of multi-sensor configurations.
- The methodology is modular and scalable, allowing for easy integration of new sensor types or mission-specific parameters without requiring a complete redesign of the system. The framework’s transparent decision logic and practical applicability make it well-suited for deployment in real-world Counter-UAV command-and-control systems.

The paper is organized as follows: Section 2 presents a concise overview of Counter-UAV Sensor technologies. Section 3 analyzes the developed fuzzy decision-making framework and its hierarchical architecture. Section 4 presents the results of the simulation-based evaluation, highlighting how the proposed fuzzy framework performs under different operational scenarios. Section 5 discusses the operational implications and limitations. Finally, Section 6 concludes the paper and outlines future research directions.

## 2. Overview of Counter-UAV Sensor Technologies

Effective Counter-UAV operations rely on a diverse set of sensing technologies, each offering distinct advantages and limitations depending on the operational context. This section provides an overview of the four primary sensor modalities commonly used in UAV detection, Radar, RF, Acoustic, and EO/IR, highlighting their core capabilities, deployment considerations, and relevance within multi-sensor fusion frameworks.

### 2.1. Radar Sensors

Radar is the most established sensor technology for aerial surveillance, traditionally deployed on elevated positions (e.g., rooftops) to provide wide-area monitoring of airspace. However, the very small dimensions of micro and mini UAVs enable them to operate within urban canyons, flying between buildings at low altitudes, which significantly reduces the effectiveness of conventional radar systems [6,24]. Line-of-Sight (LoS) interruptions caused by surrounding structures often prevent detection, while small UAVs present low radar cross sections (RCS) and low velocities, further complicating identification [6,7]. A novel concept is proposed in [24], which uses the distributed low-cost Commercial-Off-The-Shelf (COTS) radars to detect the small drones and create continuous coverage in a highly urbanized environment.

One of radar’s most important characteristics for UAV detection is the exploitation of micro-Doppler (m-D) signatures, which arise from the rotation of propellers or other moving parts of the UAV. These features allow not only classification of UAV type but also discrimination from other moving objects such as birds or aircraft, typically through statistical analysis or machine learning applied to extracted m-D features [25]. Compared to other sensing modalities, radar provides long-range detection and operates independently of weather conditions [12]. Nevertheless, its performance against very small, slow, and low-flying UAVs remains limited, and effective use requires trained operators in the absence of automation [6,7,12].

Recent research has focused on specialized radar architectures and advanced signal processing techniques developed specifically for UAV detection. These include holographic and multi-static radar approaches, as well as the integration of machine learning classifiers that exploit m-D signatures to improve automatic recognition of small UAVs in cluttered environments. Such advancements significantly enhance radar’s role as a primary detection layer in multi-sensor Counter-UAV systems [24,25,26].

### 2.2. RF Sensors

Radio-frequency (RF) detection involves monitoring and analyzing the signals transmitted between a UAV and its ground control station, aiming to identify active communication links used for command, telemetry, and payload transmission (e.g., video or images). Most commercial UAVs operate in the 2.4–5.8 GHz bands, with ranges of several kilometers, typically requiring LoS between the UAV and controller [7,10,12].

RF detection offers several advantages: it is comparatively low-cost, unaffected by weather or lighting conditions, and uniquely enables the localization of both the UAV and its ground control station [10]. When used in passive mode, it does not require regulatory licensing. However, its effectiveness depends on knowledge of the communication protocols used between UAVs and ground stations, which vary widely across manufacturers [12]. As UAV production expands, maintaining updated protocol libraries becomes increasingly challenging.

Further limitations include a relatively short operational range and a reliance on LoS between the sensor and the UAV or controller [7,12]. Performance is particularly degraded in urban environments due to congestion in Wi-Fi bands (2.4/5.8 GHz), which are heavily used by domestic networks, resulting in signal interference and false alarms. Importantly, RF detection is not effective against fully autonomous UAVs that rely on satellite navigation (GNSS), inertial navigation systems, or cellular control links instead of conventional radio communications [2,7,12].

Recent research emphasizes the importance of RF sensing within multi-sensor C-UAV architectures, particularly when fused with radar or EO/IR, where RF serves as a first-line passive detection mechanism. Machine-learning-based RF fingerprinting and advanced spectrum analysis are emerging techniques to improve robustness against interference and protocol variability [10,11].

### 2.3. Acoustic Sensors

Acoustic detection relies on recognizing the characteristic sound signatures generated by UAV propellers and motors during flight, typically captured by arrays of electric microphones and compared against reference libraries of acoustic signatures [7,8,25,27]. This method can detect UAVs even when they operate autonomously without active radio links. Additionally, by using microphone arrays, it is possible to estimate the UAV’s position through triangulation. Acoustic sensors are cost-effective and can complement other modalities such as radar, especially for low-altitude UAVs in urban areas.

However, their range is limited to approximately 300 m under suitable conditions, and performance degrades significantly in noisy environments, high wind, or high humidity [6,7,8]. Detection accuracy is also reduced when the UAV’s acoustic signature is not included in the reference library, or when applied to fixed-wing UAVs with engines off. These limitations make acoustic sensors most useful in short-range, supportive roles rather than as standalone systems.

From an operational perspective, large-scale deployment of microphone arrays in airports, energy facilities, or urban centers introduces additional challenges. Synchronization of a large number of microphones requires dedicated equipment, and shielding against electromagnetic interference is necessary to ensure accurate measurements. Furthermore, the transmission of raw acoustic data demands extensive use of recorders, switches, and cabling, increasing both system complexity and cost.

Recent research has focused on overcoming these challenges through advanced signal processing and machine learning [25]. For instance, Sedunov et al. [8] demonstrated UAV detection using passive acoustic arrays. More recently, Fang et al. [9] showed that fiber-optic acoustic sensors can extend detection capabilities while reducing electromagnetic susceptibility. Other studies highlight the potential of convolutional neural networks (CNNs) applied to acoustic spectrograms for improved classification of UAV types, even under noisy urban conditions [25]. These advancements suggest that acoustic sensors, while limited in range, are evolving into reliable components of multi-sensor Counter-UAV architectures [28].

### 2.4. EO/IR Sensors

Passive electro-optical (EO) and infrared (IR) sensors enable close-range recognition and tracking of UAVs via visual and thermal imagery. EO cameras function well in daylight, while IR imaging facilitates detection under low-light or night conditions. However, when used alone for detection, these sensors suffer from environmental factors such as clouds, fog, and dust, and require illumination at night [3,25].

IR systems are more resilient to weather than visible-spectrum cameras and are more cost-effective than radar, but their detection range remains limited due to the fact that they produce lower resolution images—reported in some cases as around 50 m for small UAVs [3]. Effectiveness also depends on the orientation of the UAV relative to the camera and the strength of its thermal emission. UAVs powered by electric motors typically exhibit weaker thermal signatures compared to internal-combustion models, due to propeller-induced cooling. Battery placement also affects thermal visibility; external batteries in airflow may reduce detectability compared to internal, enclosed batteries [29].

In urban environments, characterized by low altitude, limited visibility, and numerous obstructions, EO/IR systems must incorporate high resolution, fast response times, and automatic target tracking. COTS EO cameras with high optical zoom are commonly used for visual verification, while gimbal-mounted EO/IR systems with thermal imaging and onboard image processing enable tracking in reduced visibility. Costs vary widely based on embedded processing, installation type, and sensor intelligence.

In line with these observations, Pereira et al. [30] highlight how decision-level fusion between EO and IR streams improves detection reliability for small, non-cooperative UAVs, even in cluttered and dynamic urban settings. Similarly, Yasmeen and Daescu [31] emphasize the necessity of robust vision-based detection architectures and comprehensive datasets tailored to low-altitude, obstructed environments, reinforcing the critical role of EO/IR systems in Counter-UAV operations.

## 3. Design of the Hierarchical Fuzzy System for Counter-UAV Sensor Fusion

This study develops a hierarchical fuzzy inference system architecture for the evaluation and selection of optimal sensor configurations in Counter-UAV applications, with a focus on urban environments. The methodology was implemented in MATLAB R2024a (MathWorks Inc., Natick, MA, USA) using the Fuzzy Logic Toolbox, enabling reproducible modelling and fine-tuning of decision-making processes. All parameters, membership functions, and rule bases were defined from documented technical specifications, published performance data, and empirical overlap analyses.

### 3.1. Hierarchical FIS Architecture

**Figure 1:**
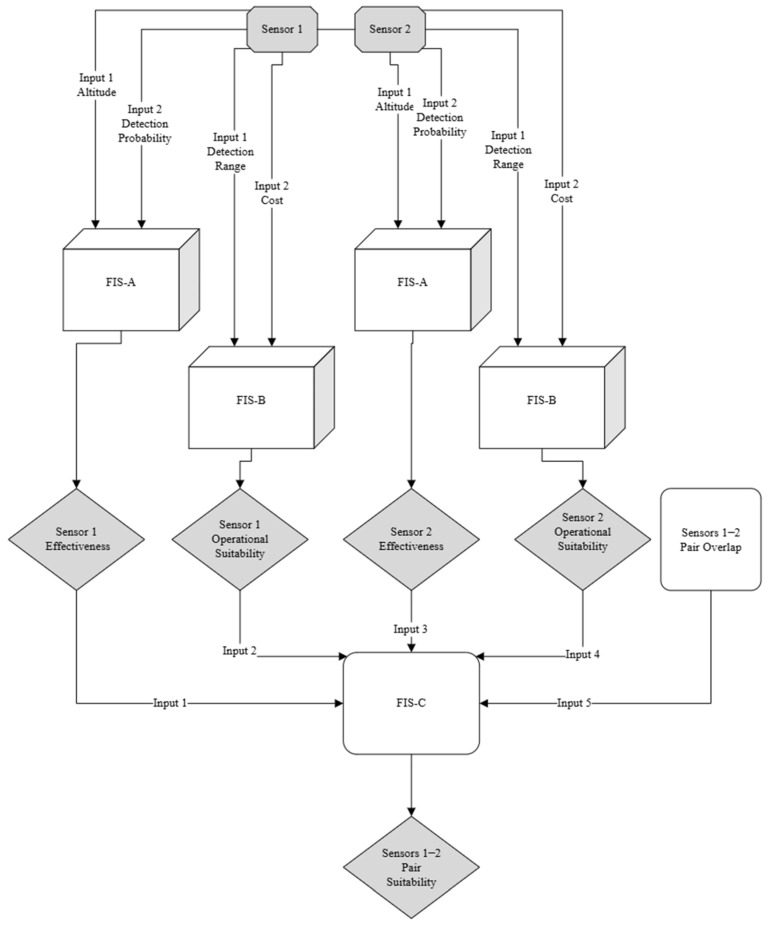
Hierarchical fuzzy decision-making architecture of the proposed Counter-UAV system.

The proposed architecture is structured into three interconnected fuzzy inference subsystems (FIS-A, FIS-B, and FIS-C; see Figure 1), each targeting a distinct decision layer in the Counter-UAV sensor selection process. This modular arrangement was adopted to manage the complexity of multi-criteria evaluation while ensuring that each decision stage remains interpretable and adaptable to evolving operational requirements.

The architecture of the system consists of three distinct subsystems. The first, FIS-A, is designed to assess sensor effectiveness using two inputs: flight altitude (0–120 m) and detection probability (0–100%), producing an output classified as low, medium, or high effectiveness. The input variables were selected based on their direct impact on detection performance in urban environments. Flight altitude directly influences a sensor’s LoS coverage, susceptibility to occlusion, and signal attenuation, all of which are critical factors in urban environments where UAVs may operate at low altitude to evade detection. Detection probability serves as the most direct quantitative indicator of a sensor’s ability to identify a target under given conditions. For each input, triangular membership functions were defined to fuzzify the crisp values, and a set of expert-derived fuzzy rules was established to map input combinations to effectiveness levels. By evaluating detection capability separately at this stage, the model avoids mixing technical performance with financial or logistical factors. This allows for fair comparisons between sensor types based only on their ability to detect UAVs.

The second subsystem, FIS-B, evaluates operational suitability using sensor range (0–2000 m) and sensor cost (0–50,000 €) as inputs, again producing an output classified as low, medium, or high suitability. Sensor range reflects the potential coverage and directly affects how many units are required for an area, thus influencing deployment feasibility. Sensor cost represents a tangible constraint in procurement and scaling, especially when safeguarding multiple facilities or large areas. Triangular membership functions were also used for these inputs, and the fuzzy rule base was constructed to reflect trade-offs between range and cost, prioritizing configurations that maximize coverage while minimizing expense. By separating operational and economic factors from raw performance, decision-makers can better understand trade-offs between performance and affordability, while also supporting scalability analysis—high-performing but expensive sensors may be most appropriate at strategic points, while more affordable options can be deployed more broadly.

The third subsystem, FIS-C, integrates the outputs of FIS-A and FIS-B for two different sensors and incorporates the pair overlap (%) metric, empirically derived from literature-based capability comparisons. The output is the overall pair suitability, classified as low, medium, or high. The inputs to FIS-C are the effectiveness and suitability scores for each sensor in the pair, along with the sensor pair overlap, which quantifies the degree of redundancy or complementarity between the two sensors. In this context, “Sensor 1” and “Sensor 2” are generic placeholders representing any two candidate sensors selected from the available pool for evaluation as a pair. The framework is designed to systematically assess the suitability of all possible sensor pairings, regardless of specific sensor type.

This stage combines technical performance and operational feasibility while adding a complementarity dimension through the sensor pair overlap input. Membership functions for the overlap input were defined based on empirical thresholds, and the fuzzy rule base was designed to reward pairs with complementary capabilities and penalize redundant combinations unless justified by high individual performance. This structure enables the optimisation of multi-sensor configurations in terms of coverage, reliability, and cost-effectiveness, while also allowing scenario-based adaptation. For example, in degraded conditions, pairs with moderate overlap may be preferred for redundancy, whereas in budget-limited scenarios, pairs with low overlap and low cost may be prioritized.

The hierarchical design prevents the rule explosion that would occur if all variables were processed within a single FIS. Each subsystem addresses a focused subset of inputs, producing interpretable intermediate outputs that feed into the subsequent stage. This layered approach improves model maintainability, as rules and membership functions can be adjusted within one stage without reconfiguring the entire system. Furthermore, the modular architecture supports incremental updates, enabling the integration of new sensor types or operational parameters into individual subsystems without the need for a complete redesign. It enhances interpretability for operators, who can understand whether a low final suitability score is driven by technical, operational, or compatibility factors. A summary of the input variables, membership function types, and rule base design for each subsystem is described in detail in Section 3.6.

### 3.2. Data Sources and Pre-Processing

The input data for the three-stage FIS framework was drawn from multiple complementary sources to ensure technical validity, real-world relevance, and cross-sensor comparability. This multi-source approach was essential because no single dataset could capture the full operational, performance, and cost spectrum of Counter-UAV sensors in urban environments.

To ensure the model was based on real-world measurements, primary performance inputs were derived from the Anti-Drone System with Multiple Surveillance Technologies study by Shi et al. [12], which tested a multi-sensor platform (RF, EO, Acoustic) against a DJI Phantom 4 in a campus environment. These empirical detection probabilities, obtained under varying distances and conditions, ensured that the fuzzy inference models were grounded in observed sensor behavior rather than relying solely on theoretical specifications.

To extend the model beyond the scope of the experimental dataset, additional performance, range, and cost figures were incorporated from peer-reviewed literature and manufacturer specifications [5,12,13,15,24,32]. This allowed the inclusion of technologies not covered in the Shi et al. study, such as radar, and enabled fair comparisons across a full suite of Counter-UAV sensors. Mixed-technology pairings were, thus, made possible, even in the absence of unified experimental trials.

To enhance the realism of the model, operational constraints commonly observed in Counter-UAV deployments were incorporated. These included environmental limitations—such as weather effects on EO/IR sensors and electromagnetic congestion impacting RF performance—as well as human-operator requirements and susceptibility to false alarms, which influence both deployment feasibility and trust in sensor outputs. Including these factors ensured that the system’s recommendations were operationally credible and not merely technical optimizations.

To prepare the data for fuzzy modeling, key variables such as Detection Probability, Range, Cost, and sensor Capability Overlap were normalized to standardized scales. This harmonization eliminated unit inconsistencies across sources, enabled comparative evaluation of different sensor types, and preserved the relative relationships observed in the literature. By combining measured data, extended specification ranges, and practical constraints, the pre-processing stage established a balanced and high-fidelity input foundation. This ensured that the fuzzy inference system could effectively model realistic trade-offs between performance, cost, and operational feasibility—an essential requirement for decision-support tools—in complex Counter-UAV scenarios.

### 3.3. Input Selection

The selection of inputs for each FIS is guided by its operational relevance, availability of reliable data, and support from existing literature. Each set of inputs was chosen to reflect the specific decision-making goals of the corresponding subsystem:

- FIS-A uses Flight Altitude and Detection Probability as input variables because both are directly linked to sensor detection performance, as supported by previous studies [11,12,26,33].
- FIS-B incorporates Sensor Range and Cost, representing key logistical and financial considerations in sensor deployment. These factors are widely discussed in the literature [5,12,13,26].
- FIS-C combines the outputs from FIS-A and FIS-B, along with empirically derived Sensor Pair Overlap (%) values. The newly introduced input quantifies the degree of redundancy or complementarity in detection coverage and is calculated using published sensor specifications, experimental data, and previous Counter-UAV systems [2,3,7,8,12,17,24,32].

### 3.4. Selection Criteria for Input Variables

To ensure the FIS accurately reflects the operational demands of Counter-UAV deployments, the input variables were selected based on a combination of practical relevance, documented evidence from the literature, and suitability for real-world implementation. Each variable was chosen to represent a key aspect of the decision-making process, helping the model maintain a balanced consideration of detection performance, coverage capabilities, cost constraints, and the complementarity between different sensor types. For instance, flight altitude has a direct and well-documented influence on detectability, particularly in dense urban environments where buildings and other obstacles introduce LoS losses, multipath effects, and visual or signal clutter. Its inclusion ensures that the model reflects realistic performance variations for sensors operating at different UAV altitudes. Detection probability represents the most direct quantitative measure of a sensor’s performance, capturing the likelihood of correctly identifying a target under specific conditions. By incorporating this variable, the system prioritizes sensors with demonstrated performance derived from measurable and observed data, rather than depending exclusively on theoretical specifications.

Sensor range determines the coverage capability of a system, influencing how many units would be necessary to monitor a given area. Longer ranges reduce deployment density, while shorter ranges may still be viable if paired with lower cost or higher detection reliability. Sensor cost is equally critical, as procurement and lifecycle expenses directly affect operational feasibility; its inclusion enables a trade-off analysis between high-performance but expensive solutions and more economical alternatives, supporting decisions under budget constraints or in large-scale deployments. Pair overlap measures the degree of functional redundancy or complementarity between two sensors. While high overlap can signal inefficient resource use unless accompanied by significant reliability gains, low overlap often indicates complementary strengths that can improve overall coverage. Including this metric ensures that the framework favors balanced sensor combinations rather than simply pairing the most capable individual sensors.

To ensure smooth output transitions and avoid abrupt shifts in suitability scores, each variable was modelled using three triangular membership functions with an approximate 25% overlap between adjacent terms. This approach provides gradual changes in output when input values vary slightly, making the system robust to noise and measurement errors. The triangular function form also enhances interpretability, as it is both visually and conceptually intuitive for operators and analysts, while remaining computationally efficient for rapid scenario evaluation. By combining these variables within a structured, fuzzified format, the model captures both the technical performance and operational realities of Counter-UAV sensor selection, ensuring adaptability across different scenarios and scalability for diverse operational contexts.

### 3.5. Input Sensor Pair Overlap

The Sensor Pair Overlap input of the FIS-C system quantifies the degree to which two sensors share similar detection capabilities, directly influencing the complementarity of their combined deployment. This variable was essential to include, as it captures a dimension beyond raw performance and cost: the interaction between sensors. Even if two sensors individually score highly in terms of effectiveness and suitability, deploying them together may not be efficient if their functional domains substantially overlap. Conversely, pairs with moderate or low overlap can provide complementary coverage, improving robustness against environmental variability and increasing system resilience.

To construct the overlap values, three operational criteria were selected based on their critical impact on Counter-UAV performance: detection range, false-alarm resilience, and weather tolerance. These criteria were chosen among a broader set of possible evaluation factors identified during the literature review, such as the ability to detect autonomous UAVs, night and day operability, ground station detection, simultaneous multi-UAV tracking, performance in congested network environments, operator requirements, licensing constraints, and cost considerations. While highly relevant, these additional factors were excluded from the current overlap calculation to preserve model parsimony and to avoid inflating the dimensionality of the FIS-C system. Nevertheless, they remain valuable dimensions of analysis and could be integrated into future research, particularly in more advanced frameworks that aim to capture the full operational, regulatory, and logistical spectrum of sensor performance.

Each sensor was evaluated using a four-level ordinal scale (★–★★★★), where ★ corresponds to 25% performance and ★★★★ to 100%. Table 1 presents the qualitative star-based assessment per sensor and criterion.

This star-based approach was chosen instead of crisp values for two main reasons. First, the literature does not provide consistent quantitative benchmarks across all four sensor types under the same environmental conditions. Attempting to unify disparate measurement frameworks (e.g., range in meters, false alarm rates in percentages, and qualitative descriptions of weather robustness) would have led to biased or incomparable values. Second, the star scale provided a concise and transparent way to synthesize findings from an extensive literature review while preserving relative differences among sensors. In effect, the star ratings acted as a “common currency” to capture comparative performance across heterogeneous data sources.

The qualitative star-based ratings were first converted into percentage values to enable quantitative comparison (★ = 25%, ★★ = 50%, ★★★ = 75%, ★★★★ = 100%). For each sensor pair and evaluation criterion, the average of the two sensors’ percentage scores was calculated. Then, the overall Sensor Pair Overlap was computed as the mean of these three criterion-specific averages. These values served as the quantitative inputs for the FIS-C system, allowing the system to assess the complementarity and redundancy of sensor combinations in a structured and interpretable way (see Table 2).

Example calculation for the Radar–RF pair:

- Detection range overlap: (100% + 75%) ÷ 2 = 87.5%
- False alarm resilience overlap: (25% + 50%) ÷ 2 = 37.5%
- Weather tolerance overlap: (100% + 75%) ÷ 2 = 87.5%
- Final average overlap = (87.5% + 37.5% + 87.5%) ÷ 3 ≈ 70.8%

By incorporating Sensor Pair Overlap as the fifth input of the FIS-C system, the framework gained the ability to distinguish between redundancy-driven inefficiencies and complementarity-driven advantages. This not only aligned the system more closely with operational practice, where sensor diversity is as important as sensor quality, but also provided a mechanism to prevent misleadingly high suitability scores for sensor pairs that are, in effect, duplicates of one another.

### 3.6. Membership Function Design

**Figure 2:**
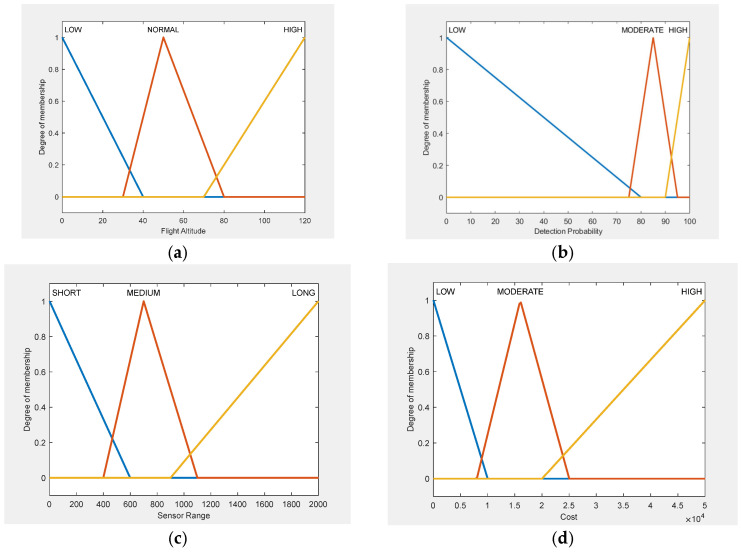
Membership functions for FIS-A and FIS-B inputs: (**a**) FIS-A input 1 “Flight Altitude” (**b**) FIS-A input 2 “Detection Probability” (**c**) FIS-B Input 1 “Sensor Range” (**d**) FIS-B Input 2 “Sensor Cost”.

**Figure 3:**
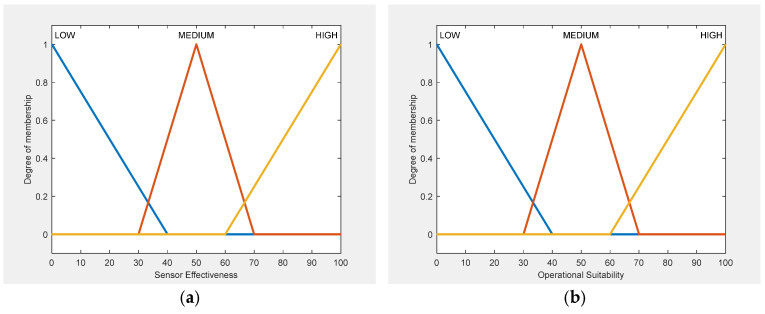
Membership functions for FIS-A and FIS-B outputs: (**a**) FIS-A output “Sensor Effectiveness” and (**b**) FIS-B output “Operational Suitability”.

The membership functions for all input variables in FIS-A and FIS-B (Figure 2) as well as for output variables (Figure 3) are designed to achieve two main goals: to reflect realistic operational behavior and to maintain computational efficiency.

The membership functions were defined using three main sources: manufacturer specifications, empirical performance data, and expert knowledge. Manufacturer specifications provided baseline values for typical Counter-UAV sensors, including detection range, operational altitude, and cost levels. These values were used to establish the universes of discourse for each input variable. Experimental detection data from peer-reviewed field studies provided critical insight into real-world behavior, including detection probabilities, range degradation, and environmental effects. This ensured that MF boundaries were anchored in observed performance, avoiding over-optimistic or overly conservative definitions. For variables lacking standardized data, such as Pair Overlap, expert-derived operational thresholds were applied; for example, “Low” overlap was set between 40 and 55%, “Medium” between 50 and 70%, and “High” between 65 and 80%, based on values observed in multi-sensor deployments rather than theoretical assumptions.

**Figure 4:**
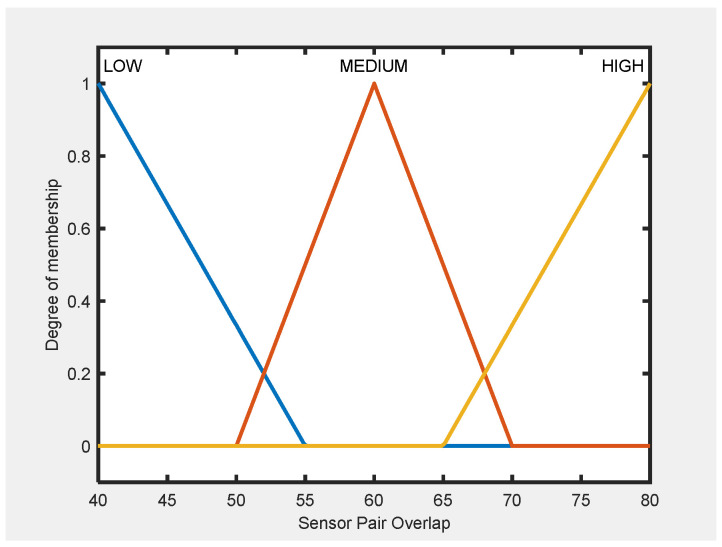
Membership function of the 5th input for FIS-C “Sensor Pair Overlap”.

**Figure 5:**
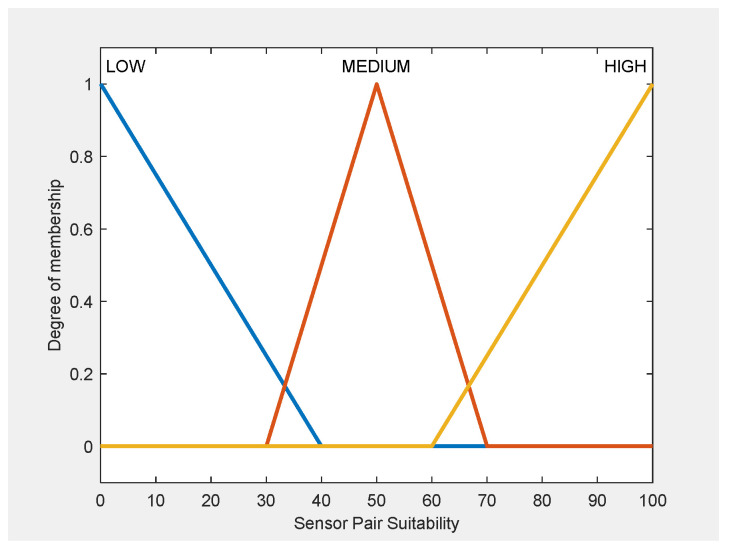
Membership function for FIS-C output: forFIS-C output “Sensor Pair Suitability”.

The membership functions for the fifth input variable of FIS-C (Sensor Pair Overlap) are illustrated in Figure 4, while the output variable is shown in Figure 5. The remaining four input variables of FIS-C are the effectiveness and suitability scores derived from FIS-A and FIS-B for each sensor in the pair. All MFs are designed to achieve two main goals: to represent sensor-pair interactions under realistic operational conditions and to ensure computational efficiency.

For FIS-C, membership functions were defined using manufacturer specifications, empirical data, and expert input, consistent with the outputs of FIS-A and FIS-B. Sensor Effectiveness and Suitability followed the same input ranges, while the Sensor Pair Overlap was modeled using expert-derived thresholds (Low: 40–55%, Medium: 50–70%, High: 65–80%), ensuring operational relevance and consistency across all subsystems.

All variables were modelled using triangular membership functions (trimf), chosen for their computational efficiency and ability to create smooth decision boundaries. Their simple geometric structure enables fast computation, which is especially useful in time-sensitive operational settings. Their shape also makes them easy to interpret, allowing operators and non-technical users to clearly understand how input values correspond to linguistic terms. Approximately 20% overlap between adjacent terms was applied to avoid hard cut-offs in the output space, ensuring that minor variations in input, such as a small drop in detection probability, would not produce disproportionately large shifts in suitability scores.

By combining real-world data, expert knowledge, and a consistent fuzzy logic approach, this MF design produces results that are both reliable for practical use and flexible enough to adapt to different operational needs. The resulting framework is robust enough for deployment in dynamic and uncertain environments, such as urban counter-UAV operations, while retaining the flexibility to incorporate future updates as sensor technologies evolve and operational priorities shift.

### 3.7. Development of the Fuzzy Rule Framework

The fuzzy inference rules for the hierarchical system were developed independently for each subsystem (FIS-A, FIS-B, and FIS-C) to ensure that the decision logic is appropriately tailored to each evaluation layer. This modular approach allows operational reasoning to be embedded at the correct level of analysis while maintaining clarity, interpretability, and flexibility. As a result, the rule base can be easily adapted to accommodate new sensor types or evolving mission requirements without compromising the overall structure of the system.

In FIS-A and FIS-B, the goal is to independently assess each sensor’s Effectiveness and Operational Suitability using the most relevant input parameters. The rules are constructed to prioritize high-performing configurations. For example, a sensor with high detection probability and suitable altitude in FIS-A, or medium range and low cost in FIS-B, will receive a high score. On the other hand, combinations with poor performance are penalized. A sensor with low detection probability or short range cannot achieve a high rating just because it is inexpensive. This approach ensures technical consistency and establishes reliable baselines before sensor pairs are evaluated.

For FIS-C, the rule base is intentionally designed to be general and independent of specific sensor types. Instead of creating separate rules for each sensor pair, a single inference logic is applied uniformly across all combinations. This approach ensures consistency and fairness in evaluating sensor pairs within a unified decision framework. The generic rules integrate four inputs (Effectiveness (A), Effectiveness (B), Suitability (A), Suitability (B)) with the empirically derived Sensor Pair Overlap input to capture complementarity. Pairs with strong individual effectiveness, balanced suitability, and low-to-moderate overlap are promoted because they widen coverage without unnecessary redundancy; conversely, high overlap is penalized unless accompanied by exceptional individual performance that justifies the added cost. To maintain operational realism, the model applies monotonic and symmetric constraints. This means that swapping the order of sensors does not affect the result, and two low-effectiveness sensors are penalized in the rule base and cannot achieve a high suitability score. These rules help avoid unrealistic recommendations and keep the outputs aligned with practical deployment standards in multi-sensor Counter-UAV systems.

This approach uses a general rule-based structure in FIS-C to ensure fairness across all sensor pairs. It avoids hidden bias that could result from pair-specific tuning. It also supports reproducibility, as new sensors can be evaluated using the same logic without the need to create custom rules.

In principle, a complete rule base with two inputs and three membership functions each would yield 9 rules per subsystem. However, in this study, the number of rules was intentionally reduced to better reflect operational evidence and the specific characteristics of each subsystem. In FIS-A. The acoustic model includes 5 rules, Radar 8, RF 6, and EO/IR 7, instead of the theoretical 9 for each case. Similarly, in FIS-B, the Acoustic subsystem includes 7 rules, the Radar subsystem uses 5 rules, while RF and EO/IR use 6 rules each. This reduction avoids unnecessary complexity and ensures that the rule sets are aligned with empirical findings reported in the literature and the realistic performance boundaries of each sensor. As a result, the models focus on capturing practical sensor performance in urban Counter-UAV scenarios, avoiding redundant or unrealistic combinations and preserving interpretability.

**Figure 6:**
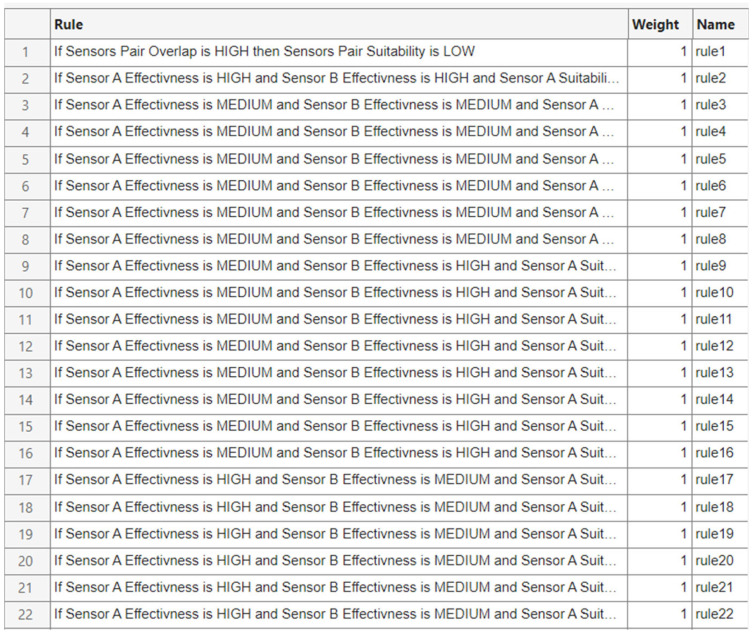
Fuzzy rules for FIS-C.

FIS-C includes a total of 32 rules, with 22 of them illustratively presented in Figure 6 to demonstrate the system’s inference logic. The initial design considered the full combinatorial space of 243 rule candidates (3^5^), which was then refined through several steps. First, structural constraints were applied to enforce symmetry between Sensor A and Sensor B (ensuring order-invariant outcomes) and monotonicity with respect to effectiveness and suitability. Unrealistic combinations, such as two Low-effectiveness sensors producing a high suitability score, were excluded. Second, complementarity was modeled using empirically defined overlap thresholds (Low: 40–55%, Medium: 50–70%, High: 65–80%), with the system rewarding low to moderate overlap unless high overlap was justified by strong individual sensor performance. Third, combinations that were not relevant to real-world Counter-UAV deployments were removed. This targeted refinement resulted in a compact, generic, and interpretable rule base that captures meaningful sensor pair interactions while avoiding rule explosion and preserving clarity.

This structure helps the hierarchical fuzzy system remain consistent across its subsystems. It also ensures that the ranking of sensor combinations is transparent and that the framework can be easily updated as operational needs or available sensors change.

### 3.8. Scenario Framework

The hierarchical FIS framework is tested under two operational scenarios that represent different deployment strategies. These scenarios were carefully selected to represent practical requirements commonly encountered in Counter-UAV systems. This approach allowed the decision-making process to be examined under varying cost and performance trade-offs, without changing the structure of the fuzzy system itself.

The first scenario represents a tactical, resource-efficient configuration, where sensors operated at close range with low procurement cost but maintained high detection probability. This case reflects deployments where affordability and rapid coverage are prioritised, such as temporary protection of small venues or urban sites. By combining short range and low cost with high detection reliability, Scenario 1 captures the conditions of a lightweight but highly effective Counter-UAV setup.

The second scenario reflected a strategic configuration, with medium-to-high sensor ranges and correspondingly higher costs, but with detection probability constrained at 90%. This threshold is selected to represent the operationally accepted minimum for reliable detection, ensuring that performance remains robust even as range and financial requirements increase. Such a scenario mirrors large-scale or long-term deployments, where investment in extended coverage is justified by the need to protect wider areas or multiple assets simultaneously.

To ensure comparability between scenarios, the Sensor Pair Overlap (%) values for each sensor pair are fixed based on empirical analysis (Section 3.5). This guarantees that observed differences stem exclusively from changes in effectiveness and operational suitability inputs, while the inherent complementarity between sensor types remains constant.

This scenario design highlights how the framework can differentiate between tactical and strategic deployment conditions. It enables controlled comparisons of cost-effective versus coverage-intensive strategies, while preserving operational realism and ensuring that sensor pair recommendations remain consistent with Counter-UAV mission requirements.

## 4. Scenario-Based Results for Counter-UAV Sensor Fusion

This section presents the results of the simulation-based evaluation of the proposed hierarchical fuzzy inference system for Counter-UAV sensor selection. The performance of the system is analyzed across its three subsystems, FIS-A, FIS-B, and FIS-C, under two distinct operational scenarios. These scenarios were designed to explore how the framework responds to different deployment conditions, focusing on variations in cost, detection range, and performance indicators. Scenario 1 represents a typical deployment setup, while Scenario 2 reflects a more demanding and resource-intensive context.

- Scenario 1 (S1): A short-range operational context with close UAV distances, high detection probability, and relatively low sensor cost. This setup reflects deployments in constrained urban environments, where low-altitude UAVs attempt to exploit LoS gaps, but detection conditions remain suitable. (FIS-A inputs: Flight Altitude = 50 m, Detection Probability = 93%; FIS-B inputs: Detection Range = 500 m, Cost = 9000 €).
- Scenario 2 (S2): A medium-to-long-range operational context with UAVs detected at extended distances, higher associated sensor costs, and a fixed detection probability of 90% (set as a realistic operational threshold). This scenario represents more resource-intensive deployments where performance must be sustained under higher logistical and financial demands, despite potential environmental challenges. (FIS-A inputs: Flight Altitude = 80 m, Detection Probability = 90%; FIS-B inputs: Detection Range = 1000 m, Cost = 15,000 €).

The analysis focuses on how sensor effectiveness (FIS-A), operational suitability (FIS-B), and sensor-pair performance (FIS-C) differ across these two scenarios. This approach allows the evaluation to capture not only detection performance but also the interaction between technical and operational factors that influence optimal Counter-UAV configurations.

### 4.1. FIS-A: Sensor Effectiveness Under Operational Conditions

FIS-A was developed to evaluate sensor effectiveness under realistic urban operating conditions by using two key input variables: Flight Altitude and Detection Probability. These inputs were selected for their direct operational relevance. Flight Altitude strongly affects LoS coverage and susceptibility to occlusion, particularly in dense cityscapes where UAVs may deliberately fly low to avoid detection. Detection Probability, meanwhile, serves as the most direct quantitative indicator of performance, capturing the likelihood of correctly identifying a UAV under given conditions. The combination of these two variables ensures that the evaluation is rooted in actual operational constraints rather than theoretical specifications.

This design offers two main advantages. First, it allows detection capability to be evaluated independently, so sensors can be compared based on technical performance without being influenced by cost or deployment factors, which are considered separately in FIS-B. Second, it provides a clear reference point for interpretation. Decision-makers can easily see whether the limitations of a sensor are due to its technical performance or to external factors such as cost or redundancy, which are addressed in later stages of the framework.

**Figure 7:**
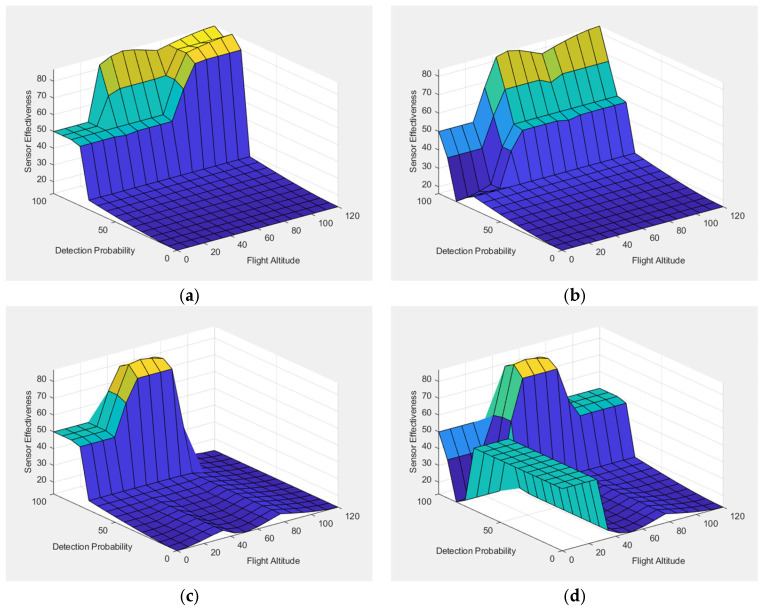
Three-dimensional surface plots of FIS-A for each sensor type, illustrating the effect of Flight Altitude and Detection Probability on overall effectiveness: (**a**) Radar; (**b**) EO/IR; (**c**) Acoustic; (**d**) RF.

Figure 7 presents 3D surface plots showing how changes in Flight Altitude and Detection Probability influence the overall effectiveness of Radar, EO/IR, Acoustic, and RF sensors. In these plots, warmer colors (yellow) indicate higher activation levels, while cooler colors (blue) represent lower activation levels. As shown in the surface plots, higher values of detection probability consistently lead to increased sensor effectiveness across all modalities. This trend highlights the sensitivity of the fuzzy system to detection performance, confirming that sensors with stronger detection capabilities are appropriately prioritized in the evaluation. The surface plots also show that flight altitude has a noticeable impact on sensor effectiveness. In general, higher altitudes tend to reduce effectiveness for sensors that rely on LoS or proximity, such as acoustic and EO/IR systems. This trend reflects the operational limitations of these technologies in detecting low-flying UAVs, especially in cluttered urban environments.

For each sensor type, the fuzzy inference process in FIS-A is applied using specific input values for flight altitude and detection probability, as defined under the two operational scenarios. These inputs are processed through the fuzzy rule base, and the resulting outputs are defuzzified to produce numerical scores representing sensor effectiveness. These scores are presented in Table 3 and allow for direct comparison of sensor performance under different operational conditions.

The results indicate clear differences between the two scenarios, showing how sensor performance shifts depending on the operational conditions. In Scenario 1, Acoustic demonstrates an effectiveness of 70.8, reflecting its strong classification capability in close-range conditions. Radar also performs at a high level with 86.5, confirming its robustness across detection contexts. RF (85.9) and EO/IR (83.1) similarly show strong effectiveness, supporting their role as reliable modalities in close operational scenarios. In Scenario 2, Radar effectiveness decreases slightly to 82.2, yet it continues to demonstrate robustness under long-range and high-altitude conditions. In contrast, RF effectiveness drops markedly (−38.2 points) to 47.7, underscoring its vulnerability to extended ranges and environmental constraints. Acoustic performance declines even more sharply (−53 points) to 17.8, confirming its very limited operational value under demanding conditions. EO/IR effectiveness decreases to 68.5, consistent with its sensitivity to reduced visibility and environmental degradation. Overall, the comparison confirms that Radar and EO/IR remain relatively stable across scenarios, while RF and Acoustic sensors exhibit pronounced sensitivity to harsher environmental conditions.

This structure of FIS-A provides a clear and operationally meaningful baseline for evaluating sensor detection effectiveness. It ensures that the following stages of the hierarchical fuzzy system—FIS-B for assessing operational suitability and FIS-C for evaluating sensor pairings—are built on a solid understanding of each sensor’s core detection capabilities.

### 4.2. FIS-B: Sensor Operational Suitability

FIS-B was developed to assess the operational suitability of each sensor type by integrating two inputs: Sensor Range and Sensor Cost. These variables were chosen because they directly influence real-world deployment feasibility. While technical detection capability is critical, decision-makers must also consider whether a sensor can provide adequate area coverage at a sustainable cost. Range reflects the extent of coverage and, therefore, the number of units required to monitor a given area, while cost reflects the financial limitations that often influence decisions about procurement and system scaling. By balancing these two dimensions, FIS-B ensures that operational planning moves beyond raw performance and into the domain of practical deployment.

The design of FIS-B provides a clear benefit to Counter-UAV evaluation: it enables decision-makers to evaluate the balance between coverage capability and financial feasibility, which are often competing factors in sensor deployment planning. For example, a long-range radar may cover wide areas with fewer units but carries a high financial burden, while a low-cost acoustic sensor may be more affordable but requires dense coverage to achieve operational effectiveness. By evaluating these factors within FIS-B, the model highlights where high-cost/high-performance sensors might be optimal for strategic locations, while low-cost alternatives could serve as scalable solutions in less demanding environments.

**Figure 8:**
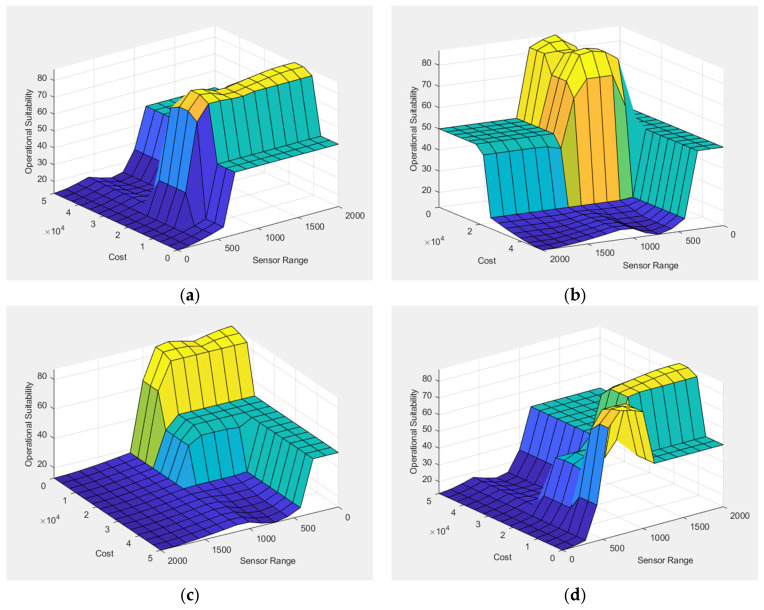
Three-dimensional surface plots of FIS-B for each sensor type, illustrating the trade-off between Sensor Range and Cost.: (**a**) Radar; (**b**) EO/IR; (**c**) Acoustic; (**d**) RF.

The 3D surface plots in Figure 8 illustrate how different combinations of sensor cost and range influence the operational suitability of Radar, EO/IR, Acoustic, and RF sensors. In general, sensors with longer detection ranges and lower costs tend to achieve higher suitability scores, as indicated by the warmer regions in the plots. This trend reflects the practical advantage of wide-area coverage at minimal expense, which is especially important in large-scale deployments. Conversely, sensors with short ranges or high costs show reduced suitability, as seen in the cooler regions. The plots also reveal that suitability does not increase linearly with range or cost; instead, the fuzzy system captures nuanced trade-offs, where moderate values may yield optimal results depending on the sensor type. These visualizations help clarify how economic and technical factors interact in shaping deployment decisions.

The defuzzified outputs corresponding to Scenarios 1 and 2 are summarized in Table 4, providing a clear comparison of each sensor’s operational suitability under different deployment conditions.

The results reveal important scenario-driven dynamics. In Scenario 1, where UAVs are detected at closer ranges and overall costs remain lower, EO/IR sensors emerge as the most suitable option (67.7), reflecting their balance between effectiveness and procurement cost in short-range detection contexts. Acoustic sensors also show high suitability (64.0), underlining their affordability and reliability at low altitudes. RF sensors achieve moderate suitability (44.3), balancing cost-efficiency with acceptable coverage, while Radar scores lowest (45.6), as its long-range advantages are not fully justified in close-range deployments.

Sensor performance shifts significantly in Scenario 2. Radar suitability rises markedly (+37.0 points) to 82.6, confirming its robustness in long-range and high-altitude detection tasks. RF sensors also improve moderately (+14.3 points) to 58.6, demonstrating their scalability under extended operational conditions. EO/IR performance increases slightly (+6.2 points) to 73.9, reflecting its enhanced role in target recognition despite higher procurement costs. Conversely, Acoustic suitability declines (–14.0 points) to 50.0, indicating reduced effectiveness under longer detection ranges and more demanding environmental constraints. Overall, Radar and EO/IR emerge as the most reliable sensors for extended-range operations, while RF and Acoustic maintain complementary but secondary roles.

By evaluating operational suitability separately in FIS-B, the hierarchical framework ensures that economic and logistical aspects are explicitly addressed. This structure allows sensors to be compared not only based on their detection capabilities, as assessed in FIS-A, but also on their practical feasibility for deployment. As a result, decision-makers are provided with a more comprehensive and realistic foundation for planning across diverse operational environments.

### 4.3. FIS-C: Sensor Pair Suitability

FIS-C represents the integrative stage of the hierarchical framework, where the outputs of FIS-A (sensor effectiveness) and FIS-B (operational suitability) are combined with empirically derived sensor-pair overlap percentages to evaluate overall pair suitability. This design is intentionally developed to align with the operational needs of multi-sensor deployments. While individual sensors may perform well on their own, real-world deployments depend on combining different sensor types. The degree to which these sensors complement or overlap with each other plays a critical role in the overall efficiency of the system. The inclusion of Sensor Pair Overlap as an input is, therefore, essential, as it quantifies how much two sensors shared similar detection domains and where they provided unique, complementary coverage. High overlap values often reflect redundancy that may not justify resource expenditure, whereas low-to-moderate overlap can reveal synergies that increase robustness and adaptability in contested environments.

This structure offers two main advantages. First, it allows sensor pairing to be evaluated through a consistent and systematic process, rather than relying on informal or ad hoc decisions. This ensures that both technical performance and operational feasibility are considered within the same decision framework. Second, it reflects real-world practices in Counter-UAV operations, where layered defense strategies often combine complementary sensor types, such as RF and EO/IR, to overcome the limitations of individual technologies under changing conditions.

**Figure 9:**
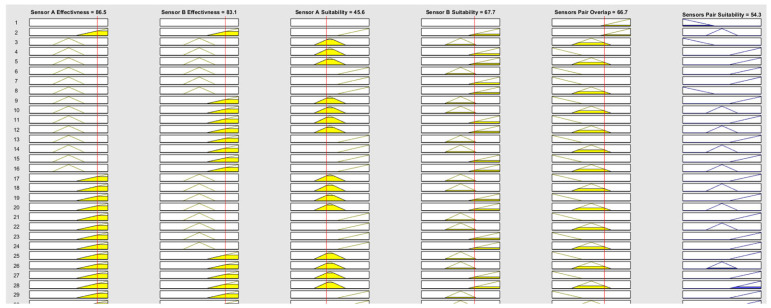
Fuzzy rule activation for FIS-C in Scenario 1.

**Figure 10:**
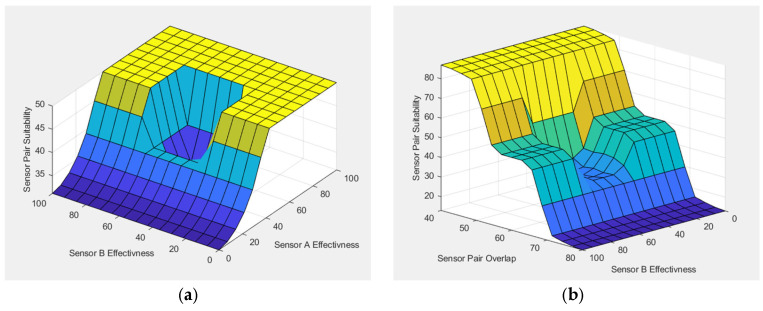
Surface plots from FIS-C showing Sensor Pair Suitability as a function of (**a**) Sensor A effectiveness and Sensor B effectiveness and (**b**) Sensor B effectiveness and Sensor Pair Overlap.

To visualize the internal reasoning of FIS-C, Figure 9 presents the activation of 29 representative fuzzy rules from the system’s rule base for Scenario 1, offering insight into how combinations of sensor effectiveness, operational suitability, and pair overlap contribute to the final suitability score. These rules exemplify the system’s logic in balancing redundancy and complementarity across sensor pairs. For instance, the input values for RADAR + EO/IR pair (Effectiveness A: 86.5, Effectiveness B: 83.1, Suitability A: 45.6, Suitability B: 67.7, and Pair Overlap: 66.7%) result in an output suitability score of 54.3. Following the rule activation visualization, indicative surface plots (Figure 10) are provided to illustrate the output landscape of FIS-C across varying input conditions, highlighting how different sensor configurations interact within the fuzzy framework.

Building on this analysis, the results of FIS-C are summarized in Table 5 and Table 6, which present the computed suitability scores for various sensor pairings under two distinct operational scenarios.

In Scenario 1, the highest suitability was achieved by the Acoustic–EO/IR pair (61.0), confirming the strong complementarity between passive acoustic sensing and visual confirmation under short-range, low-cost conditions. This combination benefits from the relatively high suitability of the acoustic sensor (64.0) and the effectiveness of EO/IR (83.1), while maintaining the lowest overlap among the pairs (54.2%). The second-best result was obtained by the RF–EO/IR pair (58.6), reflecting the operational advantage of combining RF detection of emissions with EO/IR imaging for identification.

Other pairs, such as Radar–EO/IR (54.3) and RF–Acoustic (54.9), also reached moderate suitability values, yet their higher overlaps (66.7% and 58.3%, respectively) limited their complementarity. The Radar–RF pair, despite both sensors demonstrating high individual effectiveness, received the lowest suitability (16.2) because of the very high overlap (70.8%), which renders the combination redundant. Overall, Table 5 highlights how the hierarchical fuzzy framework emphasizes complementarity over simple aggregation, rewarding heterogeneous pairs while penalizing redundant ones.

Under more demanding conditions in Scenario 2, the performance landscape shifts, revealing different strengths among sensor pairings. The Radar–EO/IR pair achieved the highest suitability (55.2), reflecting the combination of Radar’s long-range detection (82.2) with EO/IR’s classification capability (68.5). This balance supports robust target detection and identification, making the pair a viable option for frontline deployment in scenarios where greater resource investment is feasible. The RF–EO/IR pair followed closely with a suitability of 53.9, demonstrating the operational benefit of combining RF’s emission-based detection with EO/IR’s strong visual confirmation. The RF–Acoustic pair and the Radar–Acoustic pair both achieved the suitability threshold of 50.0, highlighting the limitations of acoustic sensing at longer ranges and the challenges of pairing sensors with differing sensitivities. The Acoustic–EO/IR pair dropped significantly to 33.5, confirming that acoustic detection loses operational relevance at higher altitudes and extended ranges. Finally, the Radar–RF pair remained the least suitable at 16.2 due to very high overlap (70.8%), which results in redundancy rather than complementarity. Overall, Scenario 2 results confirm that the hierarchical fuzzy framework penalizes redundant or degraded combinations while highlighting robust, complementary sensor pairings under demanding conditions.

In summary, the inclusion of FIS-C extends the framework beyond individual sensor evaluation by capturing the operational trade-offs and complementarities that emerge when sensors are paired. The benefit of this approach lies in its ability to recommend sensor combinations that are both technically robust and operationally feasible, adapting to different environmental conditions while maintaining transparency and interpretability. This ensures that the fuzzy inference system not only optimizes the performance of individual sensors but also supports system-level decision-making, which is essential for deploying reliable and cost-effective Counter-UAV solutions.

### 4.4. Comparative Analysis with Alternative Approaches

To strengthen the evaluation of the proposed hierarchical fuzzy framework, two methods were implemented for comparison: a weighted-sum model and a single-layer FIS. By applying all three methods to the same scenarios and input data, the analysis enables a direct comparison of their ability to discriminate between sensor pairs, account for redundancy, and adapt to scenario-specific conditions.

The weighted-sum method is a conventional multi-criteria decision-making approach that aggregates normalized sensor criteria using fixed weights. For each sensor pair, the suitability score is calculated as a linear combination of detection probability, range, and cost. In this study, weights were assigned, and all input variables were normalized to ensure comparability.

The single-layer FIS applies fuzzy logic to combine sensor criteria into a single suitability score. Inputs include altitude, detection probability, range, cost, and pair overlap, each mapped to linguistic terms (e.g., low, medium, high) using triangular membership functions. The output is defuzzified to yield a numerical suitability score.

Table 7 and Table 8 summarize the comparative results for sensor pair suitability of the three approaches: the weighted sum method, single-layer FIS, and hierarchical FIS for Scenarios 1 and 2. The weighted-sum method consistently produced high scores across all sensor pairs, with very limited variation (range = 5.9 in Scenario 1 and 11.4 in Scenario 2). This demonstrates its tendency to overestimate overall suitability by allowing strong criteria (e.g., detection probability) to compensate for weaknesses such as cost or overlap. The single-layer FIS, by contrast, produced mid-range scores, smoothing differences among sensor pairs due to its compressed rule base. While more advanced than the weighted-sum approach, it failed to capture scenario-driven performance shifts and penalize redundancy effectively.

The hierarchical fuzzy approach exhibited the widest dynamic range (44.8 in Scenario 1, 39.0 in Scenario 2), providing sharper discrimination between sensor pairs. In Scenario 1, Acoustic–EO/IR (61.0) and RF– EO/IR (58.6) were highlighted as the most effective combinations under close-range, low-cost conditions. In Scenario 2, Radar–EO/IR (55.2) and RF–EO/IR (53.9) emerged as the strongest pairs, reflecting their robustness under demanding conditions. The consistently low suitability of Radar–RF (16.2 in both scenarios) demonstrates the framework’s ability to penalize redundant combinations with excessive overlap (70.8%).

In addition, the hierarchical framework embeds operational knowledge through the separate rule bases of FIS-A and FIS-B, which capture sensor-specific characteristics such as effectiveness, cost, and operational suitability. This structure ensures that each sensor’s real-world constraints are explicitly incorporated into the decision process before pairwise evaluation is performed in FIS-C, thereby strengthening the interpretability and operational credibility of the results.

An illustrative example is provided by the Acoustic–EO/IR pair. In Scenario 1, this combination achieves the highest suitability (61.0), reflecting strong complementarity under close-range conditions. In Scenario 2, however, its suitability drops sharply to 33.5 because the acoustic sensor’s effectiveness decreases dramatically at longer ranges. The hierarchical framework captures this loss of complementarity and reduces the overall suitability score accordingly. By contrast, the single-layer FIS shows only a moderate decline from 44.8 to 36.0, and the weighted method remains almost unchanged from 63.6 to 62.9, since their single-layer or compensatory structures dilute or ignore the acoustic sensor’s degradation. This case study illustrates how the hierarchical approach more accurately reflects real-world operational dynamics and ensures that sensor pairings remain genuinely complementary.

Overall, the comparison confirms that the hierarchical fuzzy method provides more operationally realistic recommendations by adapting to environmental conditions, emphasizing complementarity. The weighted sum and single-layer FIS methods, while useful as baselines, do not capture the complexity and requirements of real-world Counter-UAV sensor fusion.

## 5. Discussion

### 5.1. Sensor Evaluation Outcomes and Methodological Insights

This study focuses on developing a modular and interpretable framework for sensor selection in Counter-UAV operations. The hierarchical FIS was chosen for its ability to model uncertainty, support multi-criteria decision-making, and provide transparent logic suitable for real-time deployment. In mission-critical environments, interpretability and adaptability are essential, making fuzzy logic a practical alternative to more complex data-driven methods.

The hierarchical fuzzy inference framework provides a structured basis for selecting optimal sensor combinations in Counter-UAV operations. The evaluation results of FIS-A and FIS-B confirm the importance of designing sensor selection systems based on both performance and operational feasibility. FIS-A shows that each sensor performs differently depending on altitude. Radar sensors perform best at higher altitudes with moderate detection confidence, while acoustic sensors are only effective at low altitudes with medium detection certainty. RF sensors demonstrate a broad operational range, while EO/IR sensors respond most accurately at mid-altitudes and high detection probabilities. FIS-B emphasizes the importance of balancing cost and performance. For example, radar systems achieved high suitability only when paired with moderate cost; otherwise, their suitability drops significantly. RF sensors provide a balanced solution for medium to long-range applications at an acceptable cost. Acoustic sensors are cost-effective but limited to short-range scenarios. EO/IR sensors are penalized at high cost and long range due to their limited applicability in typical Counter-UAV urban operations.

Conventional methods for sensor fusion, such as weighted-sum and single-layer fuzzy logic systems, typically lack the flexibility and depth needed for effective sensor selection in complex Counter-UAV environments. These approaches are often unable to represent the multi-dimensional trade-offs and interactions between sensors, resulting in reduced adaptability, interpretability, and scalability. As operational requirements and sensor configurations become more dynamic and diverse, such traditional systems are less capable of providing accurate and context-sensitive recommendations compared to the proposed hierarchical fuzzy inference system.

The combined use of FIS-A and FIS-B provides a multi-dimensional view of sensor performance, helping operators avoid misjudgments based solely on technical capability when deployment feasibility is limited. The modular nature of the system enables flexible decision-making, allowing it to adapt to changing mission requirements and operational conditions.

By adopting a hierarchical fuzzy inference architecture, the proposed framework overcomes these limitations by decomposing the decision process into manageable layers, each focused on a specific aspect of sensor evaluation. This structure not only prevents rule explosion but also enhances interpretability and maintainability, allowing for incremental updates and integration of new sensor types or operational parameters without a complete system redesign.

The hierarchical fuzzy inference system proposed in this study is designed to be computationally efficient and suitable for real-time applications. Each subsystem (FIS-A, FIS-B, and FIS-C) operates with a limited number of input variables and triangular membership functions, resulting in a manageable rule base and rapid inference cycles. For typical sensor configurations, the total number of fuzzy rules remains moderate, preventing rule explosion and ensuring low computational overhead. The modular architecture allows for parallel processing of subsystems and incremental updates, further enhancing efficiency. The framework can be implemented on standard computing hardware and integrated as a decision-support module within existing Counter-UAV command-and-control systems. It is compatible with common data interfaces and can operate alongside other surveillance and detection modules without requiring major changes to system infrastructure. These characteristics support practical deployment in operational environments where timely and adaptive sensor selection is critical.

### 5.2. Scenario-Based Evaluation, Limitations, and Future Directions

The comparative evaluation of Scenarios 1 and 2 demonstrates that the framework effectively captures changes in sensor performance resulting from operational constraints, such as flight altitude, detection probability, cost, and empirical sensor overlap. These outcomes directly address the limitations of single-sensor reported in previous studies [3,4,5,7,12].

The findings reaffirm earlier studies that highlighted the complementary nature of different sensing modalities [3,4,12], with radar and RF systems excelling in early detection, EO/IR being valuable in visual confirmation, and acoustic sensors providing passive coverage in RF-denied environments [12,15]. The proposed FIS-C layer proved particularly effective in modeling these complementarities, as it integrates both quantitative performance metrics (effectiveness and suitability) and qualitative assessments (overlap) to rank sensor pairs. This approach supports multi-criteria decision-making and enhances operational realism by excluding sensor pairings that are physically infeasible.

While the overlap metric was intentionally designed for simplicity and computational efficiency, it does not currently account for all operationally relevant factors, such as false alarm rates, environmental noise, night/day operability, or autonomous UAV detection. Including these additional dimensions in future work could provide a more comprehensive assessment of sensor complementarity and further strengthen the robustness of the framework.

The membership functions and scoring criteria can be adapted or recalibrated as new empirical data or operational requirements become available, allowing the framework to remain flexible and applicable to diverse scenarios. Developing more data-driven or adaptive approaches for membership function design is also identified as an important direction for future research.

The scenario-based evaluation further illustrated that environmental and operational shifts can significantly alter the ranking of optimal sensor combinations. For example, in Scenario 1, Acoustic–EO/IR (61.0) emerged as the most effective pairing under close-range, cost-sensitive conditions, highlighting the complementarity of passive acoustic and EO/IR sensing, while in Scenario 2, Radar–EO/IR achieved the highest suitability (55.2). These findings align with studies emphasizing the adaptability of sensor fusion strategies to specific mission contexts [4,12]. This adaptability is crucial for urban operations, where occlusion, multipath effects, and RF interference are frequent challenges.

From a practical perspective, the framework’s transparent decision logic and modular structure make it well-suited for integration into broader Counter-UAV command-and-control systems. This design aligns with current operational practices that emphasize flexible and scalable architectures, allowing systems to quickly adapt to evolving threat conditions.

Although experimental validation using real-world sensor data has not yet been conducted, the evaluation relies on simulation and empirical data from published sources. Future work will focus on acquiring and testing with operational sensor datasets to further validate the framework and demonstrate its effectiveness in practical deployments. In addition, future work will explore hybrid approaches and benchmarking against data-driven models, such as machine learning and Bayesian inference, as suitable datasets become available.

## 6. Conclusions

This work proposed and validated a hierarchical FIS framework for the systematic evaluation and selection of sensor combinations in Counter-UAV applications. The framework integrates three subsystems: FIS-A for operational effectiveness assessment, FIS-B for operational suitability evaluation, and FIS-C for overall pair suitability calculation using empirical overlap values derived from literature and operational experience.

Two operational scenarios were analyzed, demonstrating the framework’s capacity to adapt sensor rankings to varying environmental and operational constraints. The results showed that optimal sensor pairings differ significantly between balanced and challenging conditions, highlighting the necessity of scenario-specific sensor fusion strategies.

To evaluate the effectiveness of the proposed framework, three key performance indicators (KPIs) were considered: detection reliability, operational adaptability, and suitability scores for sensor pairings. These KPIs reflect practical requirements for Counter-UAV sensor fusion, where systems must perform consistently across varying conditions, adapt to mission-specific constraints, and recommend sensor combinations that balance performance, cost, and complementarity. The results obtained from the hierarchical fuzzy inference system demonstrate that the framework meets these KPIs, producing high suitability scores across both scenarios and effectively capturing trade-offs between sensor technologies.

The proposed methodology offers a transparent, modular, and adaptable decision-support tool for system integrators, enabling data-driven choices that account for both quantitative and qualitative sensor characteristics. The exclusion of infeasible sensor combinations in the decision-making process ensures operational realism, while grounding the overlap metrics in empirical data enhances the practical relevance of the model.

Future work will focus on experimental validation of the framework using real-world sensor data, as well as extending its capabilities for dynamic, real-time applications. These steps will be essential for demonstrating the practical value and robustness of the proposed approach in operational Counter-UAV scenarios.

## Figures and Tables

**Table 1:** Qualitative assessment of sensor capabilities for determining Sensor Pair Overlap.

Sensor Type/Criterion	Radar	RF	Acoustic	EO/IR
Detection range	★★★★	★★★	★	★★
False alarm resilience	★	★★	★★★	★★★★
Weather tolerance	★★★★	★★★	★★	★

**Table 2:** Computed sensor pair overlaps based on averaged criterion scores.

Sensor Pair	Detection Range avg (%)	False-Alarm avg (%)	Weather avg (%)	Sensor Pair Overlap (%)
Acoustic-EO/IR	37.5	87.5	37.5	54.2
RF-Acoustic	50.0	62.5	62.5	58.3
Radar-Acoustic	62.5	50.0	75.0	62.5
RF-EO/IR	62.5	75.0	50.0	62.5
Radar-EO/IR	75.0	62.5	62.5	66.7
Radar-RF	87.5	37.5	87.5	70.8

**Table 3:** FIS-A results for all sensors in Scenario 1 and Scenario 2.

Sensor	Effectiveness S1	Effectiveness S2
Radar	86.5	82.2
RF	85.9	47.7
Acoustic	70.8	17.8
EO/IR	83.1	68.5

**Table 4:** FIS-B results for all sensors in Scenario 1 and Scenario 2.

Sensor	Suitability S1	Suitability S2
Radar	45.6	82.6
RF	44.3	58.6
Acoustic	64	50
EO/IR	67.7	73.9

**Table 5:** Scenario 1—FIS-C results.

Sensor Pair	Sensor A Effectiveness	Sensor B Effectiveness	Sensor A Suitability	Sensor B Suitability	Sensor Pair Overlap	Rule Inference Pair Suitability
Radar-RF	86.5	85.9	45.6	44.3	70.8	16.2
Radar-Acoustic	86.5	70.8	45.6	64	62.5	55
Radar-EO/IR	86.5	83.1	45.6	67.7	66.7	54.3
RF-Acoustic	85.9	70.8	44.3	64	58.3	54.9
RF-EO/IR	85.9	83.1	44.3	67.7	62.5	58.6
Acoustic-EO/IR	70.8	83.1	64	67.7	54.2	61

**Table 6:** Scenario 2—FIS-C results.

Sensor Pair	Sensor A Effectiveness	Sensor B Effectiveness	Sensor A Suitability	Sensor B Suitability	Sensor Pair Overlap	Rule Inference Pair Suitability
Radar-RF	82.2	47.7	82.6	58.6	70.8	16.2
Radar-Acoustic	82.2	17.8	82.6	50	55.2	50
Radar-EO/IR	82.2	68.5	82.6	73.9	66.7	55.2
RF-Acoustic	47.7	17.8	58.6	50	58.3	50
RF-EO/IR	47.7	68.5	58.6	73.9	62.5	53.9
Acoustic-EO/IR	17.8	68.5	50	73.9	54.2	33.5

**Table 7:** Scenario 1—Methods Comparison.

Sensor Pair	Weighted-Sum Method	Single-Layer FIS	Hierarchical FIS
Radar-RF	69.0	28.2	16.2
Radar-Acoustic	65.6	45.0	55.0
RF-EO/IR	67.3	50.0	58.6
Radar-EO/IR	69.9	41.5	54.3
RF-Acoustic	63.1	50.0	54.9
Acoustic-EO/IR	63.6	44.8	61.0

**Table 8:** Scenario 2—Methods Comparison.

Sensor Pair	Weighted-Sum Method	Single-Layer FIS	Hierarchical FIS
Radar-RF	71.9	28.2	16.2
Radar-Acoustic	65.6	28.6	50.0
RF-EO/IR	69.2	49.0	53.9
Radar-EO/IR	73.1	41.5	55.2
RF-Acoustic	61.7	49.1	50.0
Acoustic-EO/IR	62.9	36.0	33.5

## Data Availability

The data presented in this study are available on request from the corresponding author.

## Abbreviations

The following abbreviations are used in this manuscript:

COTS	Commercial-Off-The-Shelf
Counter-UAV	Counter-Unmanned Aerial Vehicle
CNN	Convolutional Neural Network
EO/IR	Electro-Optical/Infrared
FL	Fuzzy Logic
FIS	Fuzzy Inference System
GNSS	Global Navigation Satellite System
KPIs	Key Performance Indicators
LoS	Line-of-Sight
MCDM	Multi-Criteria Decision-Making
m-D	micro-Doppler
MF	Membership Function
RCS	Radar Cross Section
RF	Radio Frequency
UAV	Unmanned Aerial Vehicle
